# Hydrochloric Acid Pretreatment of Different Types of Rice Husk Ash Influence on the Properties of Cement Paste

**DOI:** 10.3390/ma13071524

**Published:** 2020-03-26

**Authors:** Jing Liu, Chunyan Xie, Chao Fu, Xiuli Wei, Dake Wu

**Affiliations:** 1 College of Engineering and Technology, Southwest University, Chongqing 400715, China; 2 Chongqing Academy of Agricultural Sciences, Chongqing 401329, China

**Keywords:** rice husk ash, amount percent, acid treatment, cement paste, compressive strength

## Abstract

When properly processed, rice husk ash (RHA) comprises a large amount of SiO_2_, which exhibits a high pozzolanic activity and acts as a good building filler. In this paper, the effects of rice husk ash content, acid pretreatment, and production regions on the compressive and flexural properties and water absorption of a cement paste were studied. The experimental results showed that the compressive strength of the rice husk ash was the highest with a 10% content level, which was about 16.22% higher than that of the control sample. The rice husk after acid pretreatment displayed a higher strength than that of the sample without the acid treatment, and the rice husk from the Inner Mongolia region indicated a higher strength than that from the Guangdong province. However, the flexural strength of each group was not significantly different from that of the blank control group. The trend observed for the water absorption was similar to that of the compressive strength. The variation in the RHA proportions had the greatest influence on the properties of the paste specimens, followed by the acid pretreatments of the rice husks. The production regions of the rice husks indicated the least influence.

## 1. Introduction

China is the largest agricultural producer, and its rice output is expected to reach 210 million tons in 2019 [1]. The national average rice husk (RH) coefficient is 0.18, which means about 37.8 million tons of RHs can be obtained from rice paddies. The improper disposal of rice husks by burning or burying them in the countryside has caused a great impact on soil and air pollution [2]. However, agro-industrial wastes are regarded as emerging biomass fuel resources, and RHs are one of the largest resources. Biomass fuels are used to generate electric or heat energy, which produce less harmful gases when using a particular device [3,4]. Rice husk ash (RHA) is a potential biomass fuel product, and it contains high-purity amorphous SiO_2_ under specific combustion conditions. It can be converted easily into value-added items to be used in cement and concretes because of its high silica content [5,6,7,8,9]. Compared with ordinary cement paste, which uses limestone, electric power, and coal resources, RHA-cement is less expensive [10,11]. Furthermore, cement pastes utilizing RHA show improved mechanical properties [12,13,14,15,16,17].

The combustion temperature and combustion duration have a great influence on the activity of rice husk ashes [18,19]. The highest activity of RHA can be obtained by burning RHs at between 600 and 800 °C for 2 h [20,21]. However, the effects of other factors on the properties of RHA-cement pastes are unknown. In this work, the influence of the RHA ratios, the acid pretreatment of rice husks, and the different types of RHA on a cement paste were studied. The replacement ratios of admixture had a great influence on the performance of the cement and concrete [22,23,24]. Considerable research in this area shows that the optimum replacement ratios of various admixtures in different building materials were different. RHA contains a high percentage of K_2_O, which may act as the “activator” during the combustion. Acid treatment of rice husks prior to combustion could remove these impurities to obtain an RHA with a larger surface area and a higher activity [25,26,27]. This study examined the influence of a higher activity RHA on cement pastes. Moreover, Waheed Khan et al. reported that the chemical composition of an RHA varies with the source due to geological, geographical and climatic conditions [28]. In our work, the rice husks from Inner Mongolia (the north of China, with temperate continental monsoon climate and sufficient sunshine) and the Guangdong province (the south of China, with tropical monsoon climate, year-round high temperatures, and rainy) were compared. The effect of these different rice husks on the strength properties of cement will be discussed.

## 2. Materials and Methods

### 2.1. Cement

Ordinary Portland cement (P·O42.5) complying with GB175-2007“generalPortlandcement” and an aggregate with a specific gravity of 2.36 were used [29]. The GB175-2007 method was followed for making the paste samples. A Q8011 high-performance water-reducing agent (Shanghai Chenqi Chemical Technology Co Ltd, Shanghai, China) with a water reducing rate of 26% was used in this paper.

### 2.2. Rice Husk Ash

The RH samples were collected from Guangzhou, China and Xingunita, Inner Mongolia, China. The impurities like the sand and gravel found in the rice husks were removed by a sifter, and then the RHs were disposed through acid pretreatment. For the acid pretreatment, about 50 g of rice husk was added to 500 mL of 0.1 M HCl and boiled in a beaker for 90 min with frequent stirring. Then, we sealed the mixed solution in a beaker. After 20 h of cooling, the supernatant liquid was decanted, and the rice husk sample was washed thoroughly with distilled water until free from acid (pH neutral). After the acid pretreatment, it was then dried at 110 °C and taken in a silica crucible. The crucible with the treated RH was heated in a SX-5-12 muffle oven (Tianjin test instrument co. LTD, Tianjin, China) at a rate of 36 °C/min to 700 °C and was kept for 2 h at this temperature. The control groups were also made as described above, but the acid was replaced with distilled water. After it was burned, it was cooled to room temperature and ground into a fine ash. 

**Figure 1:**
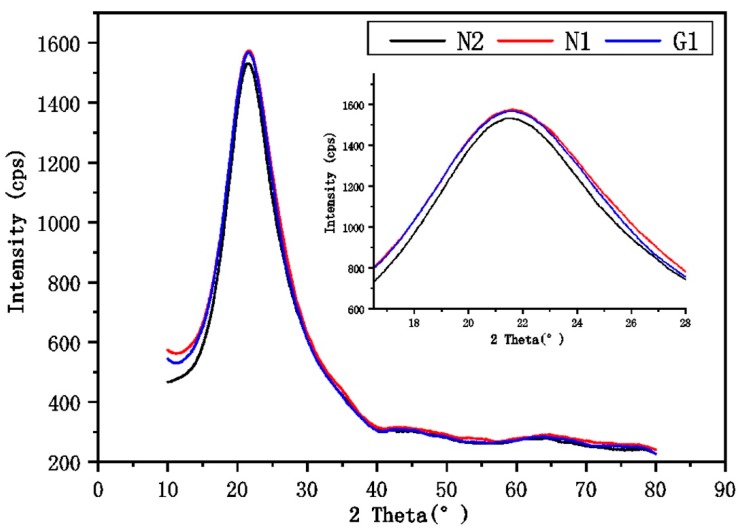
XRD patterns of the Inner Mongolia autonomous region and treated by acid (N1), Inner Mongolia autonomous region and used without acid treatment (N2), and Guangdong province treated by acid (G1) rice husk ash (RHA) samples.

The XRD patterns for the three RHA samples after the smoothing process are shown in Figure 1, and no crystalline phases seemed to occur in the ash. The typical broad hump diffraction peaks in the range of between 2θ = 12° and 40° with a maximum around 22°, which implies the presence of a high number of amorphous phases of silica in all RHAs. The upper right corner image in Figure 1 exhibits a magnified imagine at 2θ 16°–28°, revealing that the gap between N1 and G1 is smaller than that between N1 and N2.

N1-RHA means that the rice husks were from the Inner Mongolia autonomous region and treated by acid. N2-RHA means that the rice husks were obtained from the Inner Mongolia autonomous region and used without acid treatment. G1-RHA represents the rice husks from the Guangdong province and treated by acid and G2-RHA denotes the rice husks from the Guangdong province and used without acid treatment.

The chemical compositions of the four different RHAs are given in Table 1. The table displays the chemical compositions of the RHA varieties with their regions and whether they underwent acid pretreatment. The chemical composition difference between whether the RHs were acid-pretreated or not is larger than in previous sources. In contrast with N2, N1 had more silica content. Thus, the pretreatment of rice husks can reduce the content of metal ions like K+ and obtain a higher content of SiO_2_. The content of silicon in the N1/N2 rice husks is slightly higher than that of the G1/G2 rice husks, respectively.

**Figure 2:**
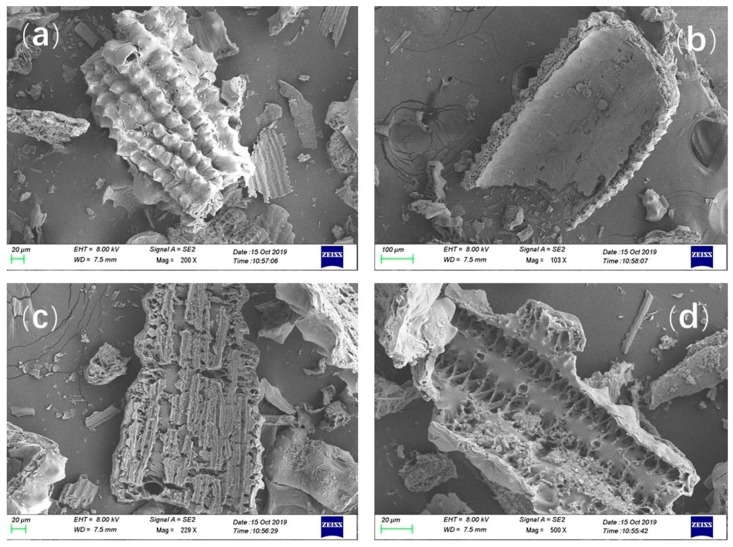
SEM images of RHA: (**a**) outer surface; (**b**) inner surface; (**c**) plane of interlayer; (**d**) cross-section of interlayer.

Figure 2 displays the SEM micrographs of the N1-RHA. The microstructure of the RHA is composed of an outer surface, an inner surface, and an interlayer. The outer surface of N1 (Figure 2a) presents a well-ranged protuberant shape while the inner surface (Figure 2b) is smooth. Figure 2c,d are a plane and a cross-section of the interlayers of the RHA, respectively, which show that the interlayer is a cellular structure which increased the specific surface area.

### 2.3. Cement Mixture Proportions

In this study, eight groups of cement paste specimens were prepared for testing, as shown in Table 2. The specimens were mixed in a mixer by mechanical stirring. The mixer conforms to the standard JG/T3033 paste mixers used for testing. The paste mixture was kept in an iron mold; it was demolded after 24 h, and then the specimen was placed into a curing box for curing for either 7, 28, or 56 days. The curing temperature was 20 ± 2 °C, and the relative humidity was 95%.

### 2.4. Testing Methods

#### 2.4.1. Compressive Strength Test

After a specified period of curing (7, 28, 56 d respectively), the eight groups of specimens, as shown in Table 2, were tested for their compressive strengths using a YAD-2000 compression testing machine (Changchun Kexin Test Instrument Co., Ltd., Changchun, China) with a 60 kN/min loading rate. Each group contained three specimens with the size dimensions of 70.7 × 70.7 × 70.7 mm. In the test data of compressive strength, if none of the three data exceed the 15% of the average, take the average as the final compressive strength value. If the maximum values or minimum values exceed 15% of the average, take the intermediate value as the final compressive strength value. If at least two values exceed 15% of the average, discard the three values.

#### 2.4.2. Flexural Strength Test

A flexural strength test was carried out on the triplicate specimens in the cement paste with the size dimensions of 40 × 40 × 160 mm, with a curing period of either 7 or 28 days. After curing, the eight group specimens, as shown in Table 2, were tested by a Dkz-5000 electric bending machine with a YAD-2000 microcomputer-controlled automatic pressure testing machine (Zhejiang Chenxi Machinery Equipment Co., Ltd., Hangzhou, China). The average flexural strength was recorded as the method in the compressive strength test.

#### 2.4.3. Water Absorption Test

After curing for 28 d, the triplicate specimens were put into a drying oven with a temperature of 105 ± 5 °C. The dry weight of the paste was recorded every 4 h until the difference between the two weights was less than 0.1% of the final mass of the specimens. The constant final mass was used as the drying mass mx_0_. After cooling to room temperature, the specimens were immersed in a water tank with steel frame-fixed specimens, and kept at the water’s surface about 20 mm higher than the specimen during soaking. After soaking for 24 h, the specimens were removed from the water and dried by a wet towel. The mass was recorded as the absorption mass mx_1_. The water absorption was calculated by Equation (1).

Wx = (mx_1_ - mx_0_) / mx_0_ × 100% (1)

#### 2.4.4. SEM Analysis of RHA Cement

An SEM analysis was carried out to study the structure of the RHA-cements at a micro level using a ZEISS Sigma 300 (Zeiss, Oberkochen, Germany). For a better observation with SEM, the dried samples were coated with gold in a sputter coater. The gold-coating was done to obtain clearer SEM images. The samples were observed at an acceleration voltage of 15 kV.

#### 2.4.5. XRD Analysis of RHA

The samples were scanned with an XRD-6100 instrument (Shimadzu, Kyoto, Japan) at a speed of 1 °/min and a range of 10–80°.

## 3. Results and Discussion

### 3.1. Compressive Strength

**Figure 3:**
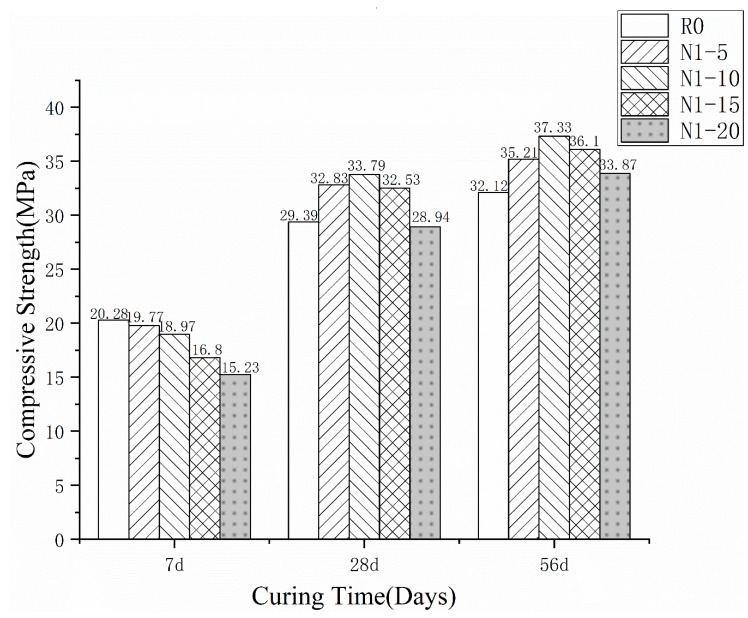
Compressive strength of different RHA rate cement at 7, 28, and 56 d.

**Figure 4:**
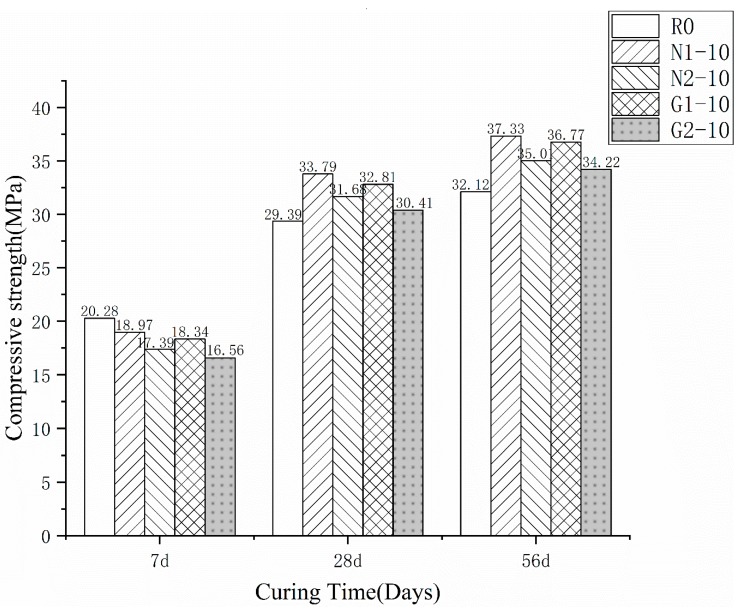
Compressive strength of different RHA cement at 7, 28, and 56 d.

The test results for the compressive strength measurements of the pastes are presented in Figure 3 and Figure 4. Figure 3 shows that the compressive strength of the pastes at different ages was influenced by the various RHA ratios. The compressive strengths of all the specimens increased with age. Furthermore, in the short period of 7 d, the strengths of the pastes were lower than the normal cement paste. The RHA has a large specific surface area and many pores in its structure. The RHA reduces the density of the cement, leading to a lower compressive strength. However, at the age of 27 d, except for N1-20, all the strengths of the specimens were higher than the normal control cement. The strengths of N1-5, N1-10, and N1-15 were 111.7%, 114.97%, and 110.68% higher than the normal cement, respectively. Moreover, the strength of N1-20 was very close to that of the control group, and the strength gap was much smaller than that at 7 d. This could be attributed to the high activity of SiO_2_ in the RHA cement; the non-crystalline or amorphous silica could react with the cement hydration products to create a C-S-H gel. The gel could fill the micropores in the cement to improve the strength. At the age of 56 d, N1-5, N1-10, N1-15, and N1-20 were increased by 9.6%, 16.22%, 12.39%, 5.45%, respectively. This suggests that the strengths of the RHA-cement pastes increased more than that of the normal paste with age. Since the overall performance of the RHA-cement paste is the best with a content of 10%, these results differ from the research by Chopra D et al., which suggested that the replacement of the 15% RHA improved the compressive and split tensile strength of the self-compacting concrete [13]. According to Alex J et al., 20 wt.% of the fine RHA addition can produce an acceptable strength of cement concrete [14]. Different materials and methods make different suitable rates of rice husk ash. In our study, a 10% RHA content was selected for the subsequent experiments. 

Figure 4 shows that the variety of rice husk affects the properties of the RHA-cement. The figure states that the compressive strength without acid pretreatment was lower than that of the treated specimens. N1-10 was lower by 7.79% of the control specimen than that of N2-10 at 7 d, and was 7.18% lower at 28 d, and 7.22% lower at 56 d. G1-10 was lower by 8.78%, 8.17%, and 7.94% of the control specimen than G2-10, respectively. This is consistent with the quantitative analysis results obtained by the XRD pattern. The RHA treated with HCl contained fewer metal impurities such as K and Mg, resulting in a higher pozzolanic activity of the RHA and a better strength performance. The pore diameter of the RHA-cement paste after acid pretreatment tends to be smaller, which may be one of the reasons for the strength increase in the cement specimen after pretreatment [30]. In addition, the strength in group N (N1-10 and N2-10) was higher than that in group G (G1-10 and G2-10). The compressive strength of N1-10 was higher by 3.11%, 3.33%, and 1.74% of the normal paste than G1-10 at 7, 28, and 56 d, respectively. The compressive strength of N2-10 was higher by 4.09%, 4.32%, and 2.46% of the normal paste than G2-10 at 7, 28, and 56 d, respectively. This indicates that the RHA from Inner Mongolia has a slightly higher compressive strength than that from the Guangdong province when added into the cement paste. Hence, for the compressive strength of the different types of RHA-cement, the influence of pretreatment on the compressive strengths of the various rice husks was greater than that of the ash from different areas.

### 3.2. Flexural Strength

**Figure 5:**
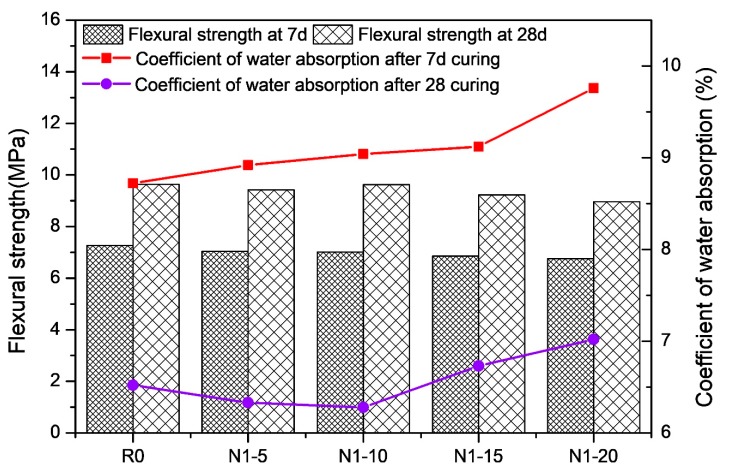
Flexural strength and coefficient water absorption of different ratios of RHA-cements at 7 and 28 d.

**Figure 6:**
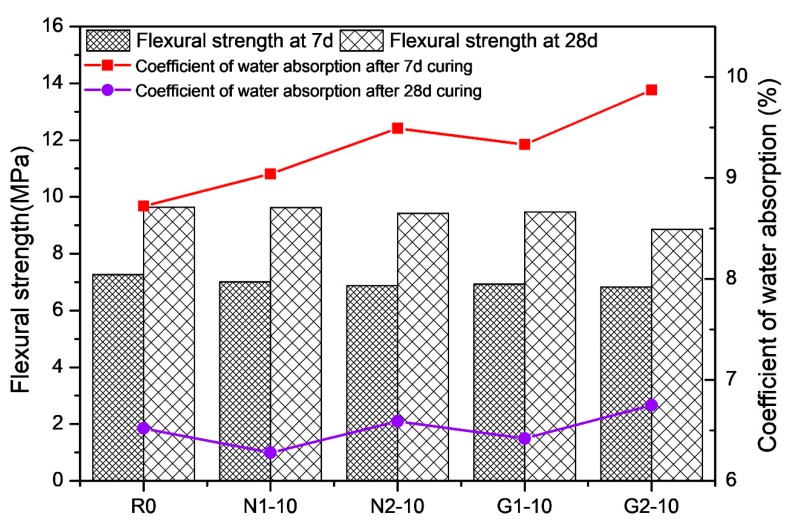
Flexural strength and coefficient water absorption of different types of RHA-cements at 7 and 28 d.

In Figure 5, the flexural strength of the RHA-cement was slightly lower than the control specimen at 7 d. This may be due to the fact that the porosity of the cement was increased when the RHA had not fully exerted its pozzolanic activity, making the cement structure looser than that of the control paste. At 28 d, the flexural strength gap between each group and the control group was very small. This could demonstrate that the flake structure of the RHA (as shown in Figure 2) did not improve the flexural strength of the cement paste, but instead slightly decreased it. The flexural strengths of the various types of RHA-cement paste are shown in Figure 6.

Figure 6 indicates that the flexural strength of the G1-10 RHA-cement was lowest at 7 d, and that the G2-10 RHA-cement was lowest at 28 d. The figure demonstrates that the different kinds of RHA had little effect on the flexural strength of the cement paste. Various contents and types of rice husk cement pastes still met the minimum requirements of the standard.

### 3.3. Coefficient of Water Absorption

All the water absorption rates of the 7 d cement pastes were higher than that of the control group, as shown in Figure 5. The water absorption and porosity improved with the increasing RHA content due to the high specific surface area of the RHA and the number of microholes in the interlayer of the RHA. At 28 d, the water absorption rate of the cement paste was much lower than that at 7 d, indicating that with the increase in age, the specimen was gradually compacted. However, N1-5, N1-10, and N1-15 were all lower than R0 in their water absorption rates; therefore, the high pozzolanic activity of the RHA during this period could be beneficial in filling the microholes that were produced before. 

Figure 6 shows the influence of the different types of RHA on the water absorption rate of the cement paste. At 7 d, the water absorption rate of the cement specimens with 10% RHA was higher than that of the blank control group R0. N2-10 posed the highest rate. At 28 d, N1-10, and G1-10 had a lower water absorption rate than the blank control group, and N2-10 and G2-10 exhibited a higher water absorption rate. Therefore, it can be concluded that acid pretreatment has an influence on the water absorption, and this influence is greater than that of the regional-origin rice husk samples.

### 3.4. Microstructure of Paste Phase Containing RHA

**Figure 7:**
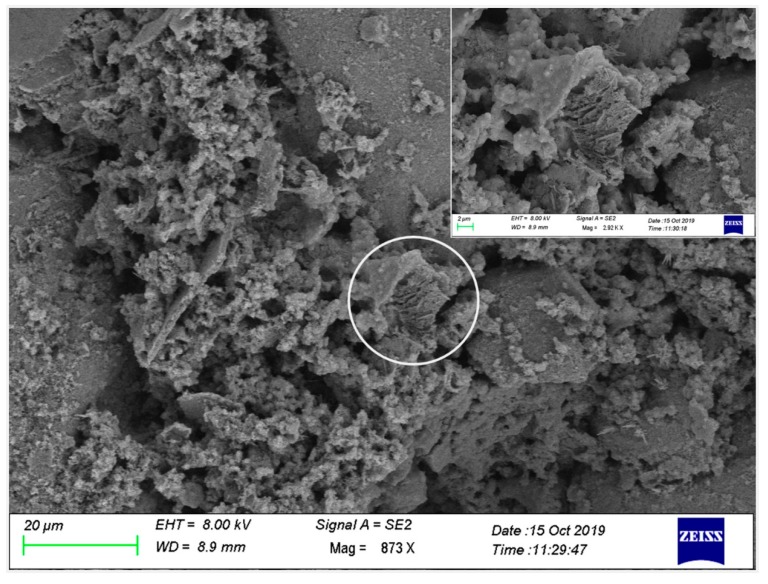
SEM view of N1-10 RHA cement at 28 d.

**Figure 8:**
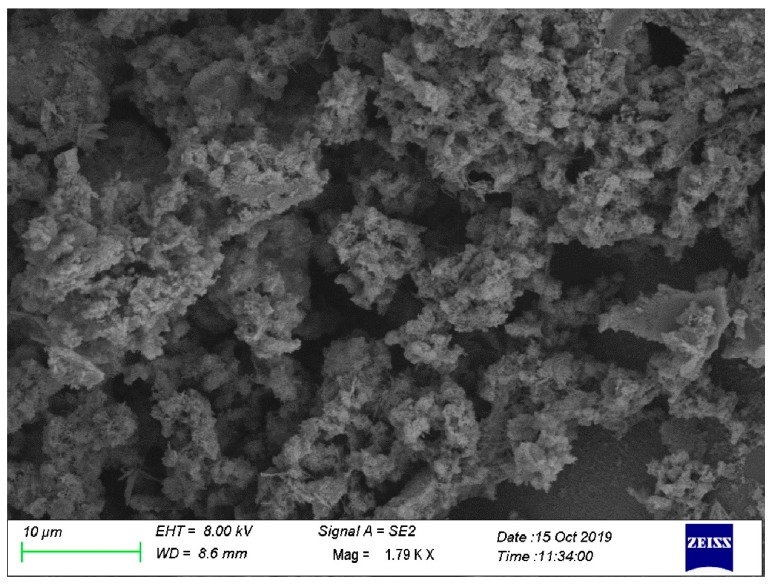
SEM view of N1-10 RHA cement at 56 d.

Figure 7 shows that calcium silicate hydrates were formed inside the cement paste at 28 d. These gel particles filled the holes in the cement paste, enabled the paste to become more compact, improved the compressive strength of the cement paste, and reduced the water absorption. The top right corner of Figure 7 is a partial enlarged view of the white circle. It can be clearly observed that the honeycomb porous structure material (RHA) was surrounded by a thick layer of C-S-H gel, which suggests that RHA has a high SiO_2_ content and a high pozzolanic activity. Figure 8 shows the SEM view of the RHA-cement paste at 56 d. In fact, the pozzolanic structure of the RHA could not be seen any more because it had reacted to generate calcium silicate hydrate.

## 4. Conclusions

1. Various contents of rice husk ash had a certain impact on the compressive strength and water absorption of the cement paste. At 28 and 56 d, except for the 20% RHA content, the compressive strengths of the other groups were slightly reduced, but all groups indicated improvement. The compressive strength of the cement was improved impressively by 16.22% at the age of 56 d when the content was 10%. The trend of the water absorption was similar to the compressive strength. Most bending strengths of the paste were lower than that of the blank, but the difference was not obvious. It could still meet the minimum strength requirements of the specification. According to the properties of the five kinds of cement paste, the content of 10% RHA was the optimal content;

2. Whether the rice husk was pretreated or not, and the origin of the rice husk also had a certain influence on the cement paste specimens. The acid pretreatment improved the compressive strength from 7% to 8%. The compressive strength of the rice husks produced in Inner Mongolia was slightly higher than that of the ash prepared in the Guangdong province; about 3% higher. The difference in the flexural strength between each group was not noticeable, indicating that the type of rice husk had little influence on the strength of the specimen;

3. The influence on the performance of the cement paste was ranked as follows: the different mixing amount that had the greatest influence, followed by acid treatment. The origin of the rice husk had the least influence;

4. There was a good agreement between the XRD patterns, compressive strengths, water absorption rates, and SEM studies of the specimens. Rice husk ash could be a promising cement or concrete admixture.

## Figures and Tables

**Table 1:** Chemical composition of different types of RHA samples.

Samples	SiO_2_	K_2_O	Na_2_O	Fe_2_O_3_	MgO	CaO	P_2_O_5_	Others
N1	97.39	0.08	0.11	0.22	0.04	0.36	0.18	1.62
N2	91.09	4.12	0.35	0.38	0.55	0.67	0.21	2.63
G1	95.27	0.11	0.19	0.14	0.21	0.33	0.37	3.38
G2	88.83	5.53	0.57	0.42	0.49	0.47	0.52	3.17

**Table 2:** Cement paste mixture proportions.

Mix	Percentage of RHA *	Origin of RHA	Acid Pretreated	Cement Content	C/S Ratio	W/B Ratio	Water Reducing Rate
R0	0	-	-	1	1/3	0.55	1%
N1-5	5%	Inner Mongolia	Yes	0.95	1/3	0.55	1%
N1-10	10%	Inner Mongolia	Yes	0.9	1/3	0.55	1%
N1-15	15%	Inner Mongolia	Yes	0.85	1/3	0.55	1%
N1-20	20%	Inner Mongolia	Yes	0.8	1/3	0.55	1%
N2-10	10%	Inner Mongolia	No	0.9	1/3	0.55	1%
G1-10	10%	Guangdong	Yes	0.9	1/3	0.55	1%
G2-10	10%	Guangdong	No	0.9	1/3	0.55	1%

* All the proportions and ratios are weight proportions.
